# Twinkling-guided ultrasound detection of polymethyl methacrylate as a potential breast biopsy marker: a comparative investigation

**DOI:** 10.1186/s41747-022-00283-z

**Published:** 2022-06-17

**Authors:** Christine U. Lee, Matthew W. Urban, A. Lee Miller, Susheil Uthamaraj, James W. Jakub, Gina K. Hesley, Benjamin G. Wood, Nathan J. Brinkman, James L. Herrick, Nicholas B. Larson, Michael J. Yaszemski, James F. Greenleaf

**Affiliations:** 1 Department of Radiology, Division of Breast Imaging and Intervention, Mayo Clinic, 200 First St, SW, Rochester, MN 55905 USA; 2 Department of Radiology, Division of Radiology Research, Mayo Clinic, 200 First St, SW, Rochester, MN 55905 USA; 3 Department of Physiology and Biomedical Engineering, Mayo Clinic, 200 First St, SW, Rochester, MN 55905 USA; 4 Department of Orthopedic Surgery, Mayo Clinic, 200 First St, SW, Rochester, MN 55905 USA; 5 Division of Engineering, Mayo Clinic, 200 First St, SW, Rochester, MN 55905 USA; 6 Department of Surgery, Division of Surgical Oncology, Mayo Clinic, 4500 San Pablo Rd, Jacksonville, FL 32224 USA; 7 Mayo Graduate School of Biomedical Sciences, Mayo Clinic, 200 First St, SW, Rochester, MN 55905 USA; 8 Department of Pharmacy, Mayo Clinic, 200 First St, SW, Rochester, MN 55905 USA; 9 Department of Quantitative Health Sciences, Division of Clinical Trials and Biostatistics, Mayo Clinic, 200 First St, SW, Rochester, MN 55905 USA

**Keywords:** Artifact, Porosity, Polymethyl methacrylate, Surface properties, Ultrasonography

## Abstract

Since its first description 25 years ago, color Doppler twinkling has been a compelling ultrasound feature in diagnosing urinary stones. While the fundamental cause of twinkling remains elusive, the distinctive twinkling signature is diagnostically valuable in clinical practice. It can be inferred that if an entity twinkles, it empirically has certain physical features. This work investigates a manipulable polymeric material, polymethyl methacrylate (PMMA), which twinkles and has measurable surface roughness and porosity that likely contribute to twinkling. Comparative investigation of these structural properties and of the twinkling signatures of breast biopsy markers made from PMMA and selected commercially available markers showed how twinkling can improve ultrasound detection of devices intentionally designed to twinkle. While this specific application of detecting breast biopsy markers by twinkling may provide a way to approach an unmet need in the care of patients with breast cancer, this work ultimately provides a platform from which the keys to unlocking the fundamental physics of twinkling can be rigorously explored.

**Supplementary Information:**

The online version contains supplementary material available at 10.1186/s41747-022-00283-z.

## Key points

- Surface roughness is associated with the color Doppler ultrasound twinkling phenomenon.
- Polymethyl methacrylate is a manipulable material with measurable surface roughness and porosity.
- Polymethyl methacrylate markers twinkle with multiple transducers using a range of ultrasound frequencies.

## Background

The twinkling artifact, described as a radiological sign, on color Doppler ultrasound [1, 2], is characterized by dynamic color fluctuations of adjacent pixels and is either empirically present or absent. Currently, there are no standards for optimizing ultrasound twinkling parameters or quantifying the twinkling signature in the clinical setting, and this is mainly because the mechanistic causes of ultrasound twinkling remain elusive. Surface roughness or irregularities and internal porosity of complicated objects have been described as contributors to the twinkling signature hypothesized to reflect phase-shift phenomena that arise in ultrasound wave propagation, interactions with bubbles or during cavitation [3–8]. In recent years, using fiducials detectable by ultrasound twinkling [9, 10] has gained some translation into the clinical setting despite not fully understanding the physics of twinkling or how to optimize the scanning parameters for generating twinkling. Seldom is clinical practice driven more by empiric evidence than by fundamental principles, but the success and inherent safety profile of color Doppler ultrasound satisfy the maxim “First, do no harm.”

Consequently, color Doppler ultrasound, which is generally not used in breast radiology could play a significant role [11] for example, when consistent and confident ultrasound detection of biopsy markers or clips is needed. Despite the availability of at least 38 commercial biopsy markers [12], ultrasound detection of these markers, particularly in treated metastatic axillary lymph nodes of patients with breast cancer, remains challenging and sometimes impossible approximately 25% of the time [13]. Color Doppler ultrasound twinkling of markers could provide a novel and specific feature for detection. Some metallic markers demonstrate a twinkling signature [9, 14], and another marker based on microsphere technology [10] also twinkles. What remains incompletely understood is why and how some markers twinkle better than others. It can be inferred that if an entity twinkles, it empirically has certain physical features.

This work investigates a manipulable polymeric material, polymethyl methacrylate (PMMA) [15] that twinkles and has measurable surface roughness and porosity that likely contribute to twinkling. Comparative investigation of these structural properties and of the twinkling signatures of breast biopsy markers made from PMMA and selected commercially available markers shows how twinkling can improve ultrasound detection of breast biopsy markers and offer a way to approach an unmet need in the care of patients with breast cancer [15].

## Methods

PMMA (Stryker Corporation, Howmedica®, Kalamazoo, MI, USA) mixed according to specifications on the package insert was made into a 1.3-mm diameter, 8-mm long cylindrical construct comparable in size to conventional biopsy markers by extruding the PMMA from a 15-gauge hole punched into the hub of a needle attached to a 3-cc syringe.

Based on earlier developed techniques [14], a non-contact three-dimensional (3D) coherence scanning interferometer optical profiler (Zygo Corporation, Middlefield, CT, USA) measured the areal surface roughness (S_a_) of four commercial metallic breast biopsy markers: TriMark® cork (Hologic®, Marlborough, MA, USA), Tumark® Q (Hologic®), UltraClip™ ribbon (Becton, Dickinson & Co., Franklin Lakes, NJ, USA), and SenoMark™ O clip (Becton, Dickinson & Co.). Optical surface characterization to measure *S*_*a*_ was performed using a consistent magnification of × 20 for all markers. Overall shape and curvature of the markers were removed from the surface characterization using 4^th^ order polynomial curve fitting of the optical measurement data [16].

Scanning electron microscopy (SEM) (Hitachi S-4700, Hitachi High-Tech in America, Schaumburg, IL, USA) images captured the surface irregularities of PMMA and the metallic biopsy markers. Porosity was determined using micro-computed tomography (SkyScan 1272, Bruker Corporation, Allentown, PA, USA) using provided computed tomography analyzer software (CTAn, Bruker Corporation) based on thresholding and regions of interest on 15-μm slice thicknesses [17].

Ultrasound of the four commercial markers and the PMMA marker was performed in a gel phantom and *ex vivo* in pork belly meat using a clinical system (Logiq E9, General Electric Healthcare, Wauwatosa, WI, USA) with 9-L and ML6-15 linear array transducers, both generally used in breast ultrasound, and a C1-6 curvilinear transducer typically used in abdominal ultrasound (General Electric Healthcare). To minimize experimental bias, the markers were placed at roughly the same depth between 1 and 2 cm deep and spaced minimally apart so that they could be scanned simultaneously. For the phantom study, two gel phantoms were stacked on top of each other to minimize backscatter from the tabletop.

Scanning parameters such as ultrasound transmit frequency, color scale, and gain were adjusted to optimize twinkling. For radiological assessment, a twinkling score was defined from 0 (least twinkling and least confident detection) to 4 (most twinkling and most confident detection) [14]. In general, a twinkling score of 3 or 4 would provide sufficient confidence for a breast radiologist to place an ^125^I seed next to it for localization without definite visualization of the marker on B-mode imaging. A twinkling score of 2 and below would require additional imaging features or information before an ^125^I seed would be used to localize it.

## Results

**Fig. 1:**
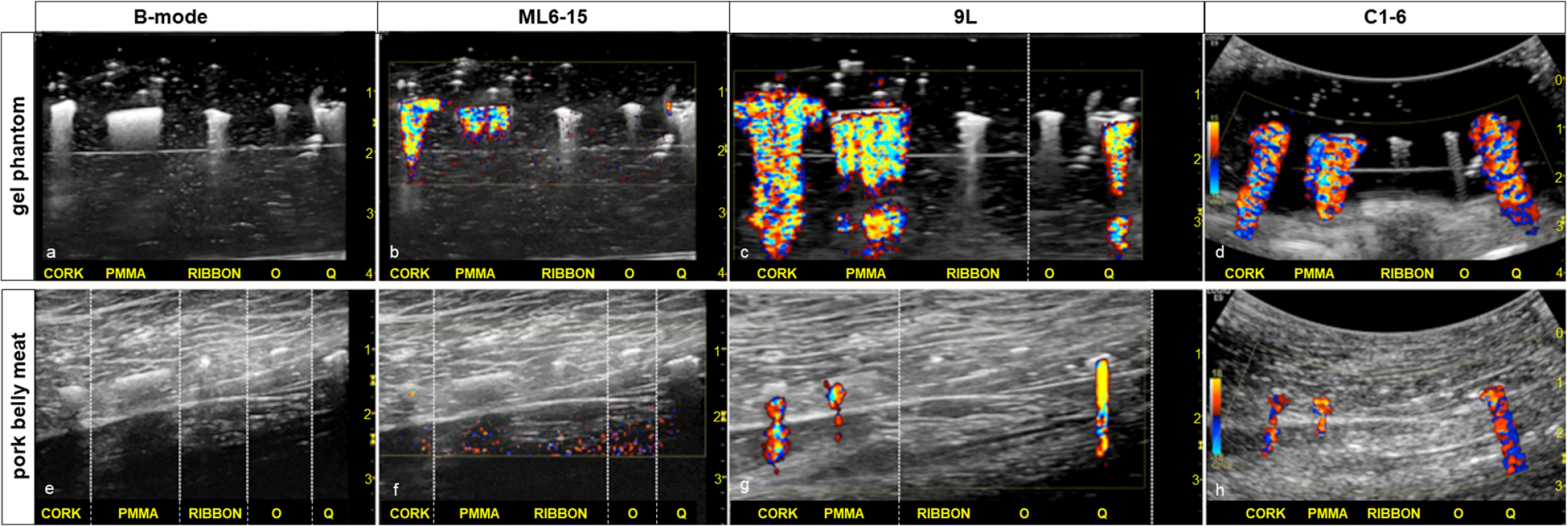
Ultrasound of five markers (cork, PMMA, ribbon, O, and Q, from left to right) are seen on B-mode imaging in a gel phantom (**a**) and in *ex vivo* pork belly meat (**e**). For the cork, PMMA, and Q markers, the ML6-15 transducer demonstrated the weakest twinkling signatures, particularly evident in the pork belly study (**b, f,** Supplemental Materials B and F). While the 9L transducer showed exuberant, persistent twinkling signatures for the cork, PMMA, and Q markers in the gel phantom (**c**, Supplemental Materials C1 and C2), the twinkling was present but reduced in the pork belly meat (**g**, Supplemental Materials G1 and G2). The C1-6 transducer shows persistent, exuberant twinkling for the cork, PMMA, and Q markers in the gel phantom (**d,** Supplemental Material D) and pork belly meat (**h**, Supplemental Material H). The ribbon and O markers had a twinkling score of 0. The five markers were not perfectly aligned along a line so could not be optimally depicted in a single image; the white dotted vertical lines (**c, e, f, g**) indicate spliced frames from the cine clip providing optimal marker visualization. The thin echogenic parallel line in the gel phantom (**a, b, c, d**) is the interface of two stacked gel phantoms to minimize back scatter from the tabletop. *PMMA* Polymethyl methacrylate


The cork, ribbon, PMMA, O, and Q markers were identified in the phantom and *ex vivo* pork belly meat by B-mode imaging using the ML6-15 transducer. With color Doppler, a distinct twinkling signature (twinkling score ≥ 3) was noted for the cork, PMMA, and Q markers. Relative to the transducers, the twinkling signature in both the gel phantom and the pork belly meat was most pronounced (highest twinkling scores) with the C1-6 transducer (color transmit frequency 3.1 MHz in the gel phantom and 3.1 MHz in pork belly meat) followed by the 9L transducer (color transmit frequency 5.0 MHz in the gel phantom and 3.1 MHz in pork belly meat), and least with the ML6-15 transducer (color transmit frequency 6.3 MHz in both the gel phantom and pork belly meat). This relationship was particularly evident in the pork belly study. Twinkling signatures that scored a 4 were evident over a range of color frequency settings towards the lower end of the spectrum for each transducer (Fig. 1 and Supplemental Materials). The ribbon and the O markers exhibited no twinkling signature (twinkling score = 0) for all transducers and all parameter settings.

**Additional file 1.**



**Additional file 2.**



**Additional file 3.**



**Additional file 4.**



**Additional file 5.**



**Additional file 6.**



**Additional file 7.**



**Additional file 8.**


**Fig. 2:**
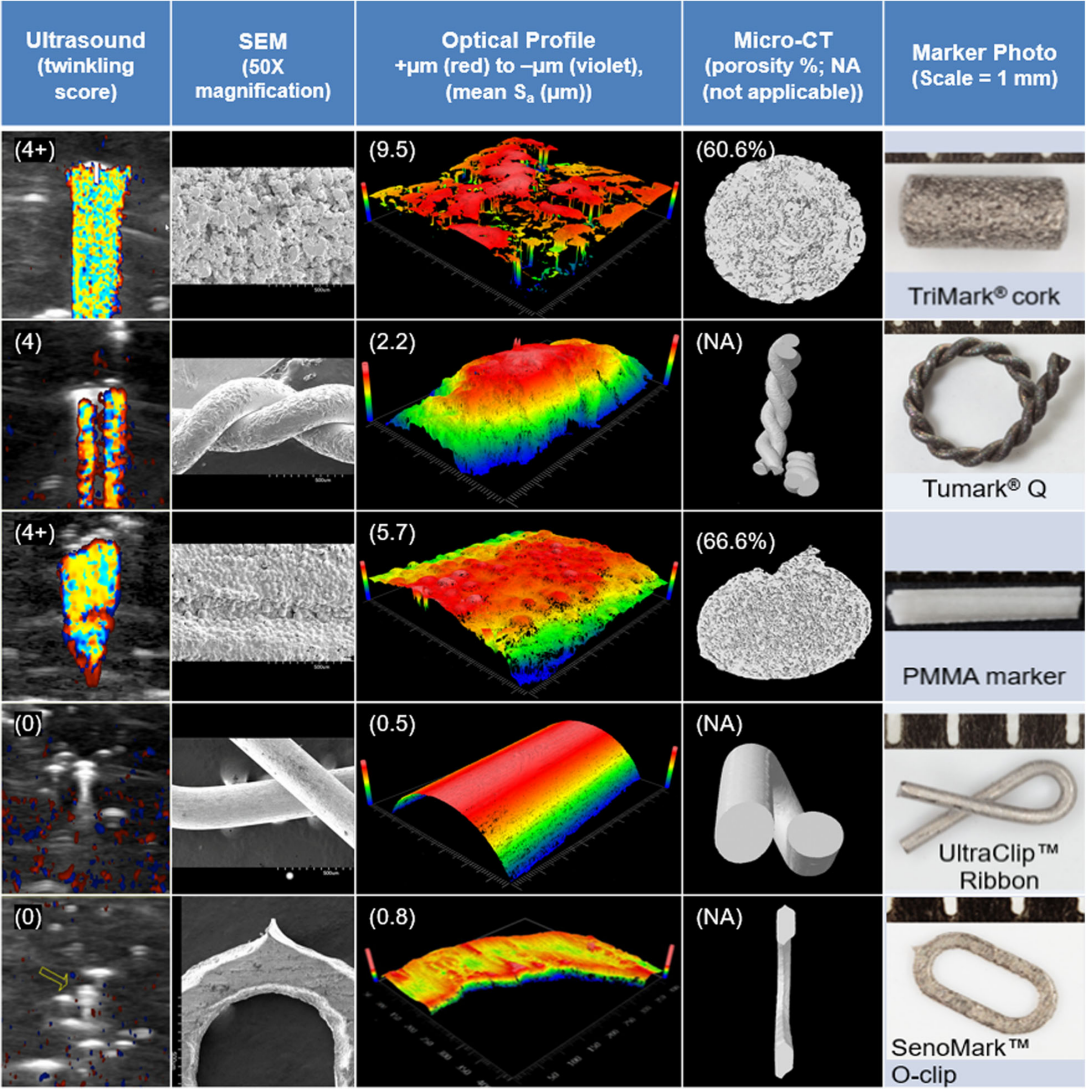
Marker characteristics on ultrasound, SEM, optical profile, and micro-CT. Color Doppler twinkling signatures of markers (1st column), and their surface features from SEM at 50 × magnification (2nd column) correlate with what was predicted by surface roughness measurements (3rd column). Additionally, micro-computed tomography (4th column) provided a metric for porosity for the TriMark® cork and PMMA markers (5th column). Based on areal surface roughness measurements (see Fig. 3), the PMMA marker could be predicted to twinkle on color Doppler ultrasound. Both the PMMA marker and the TriMark® cork were rated as 4+ twinkling, surpassing expectations. A frequently used marker, the UltraClip™ ribbon clip, was rated 0 twinkling and did not have appreciable surface roughness. *PMMA* Polymethyl methacrylate, *SEM* Scanning electron microscopy

**Fig. 3:**
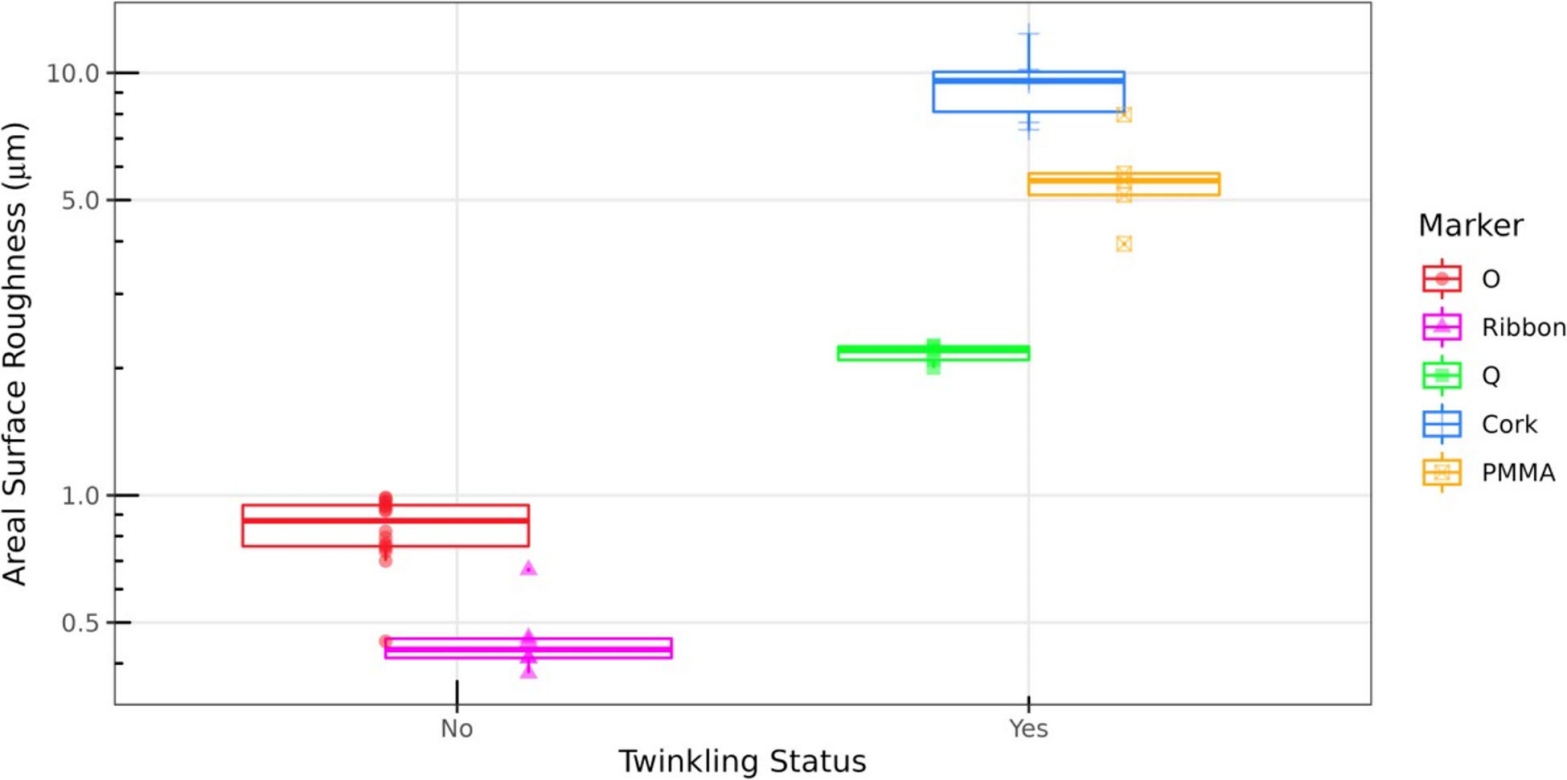
Surface roughness *versus* twinkling. Boxplots of areal surface roughness (*S*_*a*_) measurements (*y*-axis, log-scale) with individual observations plotted, grouped by consensus absence (twinkling score 0) or presence of twinkling (score ≥ 3). Boxplots are slightly jittered horizontally solely to avoid direct overlap. Coloration and plotting symbols are separately designated for each marker per the legend. Figure produced using R v3.6.1 (R Foundation). *Cork* TriMark®, *O* SenoMark™, *PMMA* Polymethyl methacrylate, *Q* Tumark®, *Ribbon* UltraClip™

The *S*_*a*_ of the commercially available cork and the Q markers was high (2−10 μm) compared to the ribbon and the O markers (< 1 μm), consistent with previously published results [14]. The *S*_*a*_ of the PMMA marker (5−6 μm) was between that of the cork and Q markers. SEM images capturing the surface irregularities of PMMA and the four metallic biopsy markers used in this study supported the *S*_*a*_ measurements. The color Doppler ultrasound twinkling signature was both present and comparable for the markers with high *S*_*a*_ (PMMA, cork, and Q markers) and essentially absent for markers with low *S*_*a*_ (ribbon and O markers) as shown in Figs. 2 and 3.

The mean porosity, as determined from micro-computed tomography, of six PMMA markers created for patient use was 66.6 ± 13.4% (mean ± standard deviation). The porosity of the cork marker (high *S*_*a*_) was 60.6%. The porosities of essentially solid wire-based constructs such as the ribbon (low *S*_*a*_) and the O (low *S*_*a*_) markers were not measurable. The exception was the Q marker (high *S*_*a*_) which had non-detectable porosity. Unlike *S*_*a*_, the association between porosity and twinkling appears weaker, favoring surface roughness as a stronger contributor to twinkling (see Fig. 2).

## Discussion

After 25 years, the fundamental causes of twinkling on color Doppler ultrasound have yet to be determined. This work investigates a manipulable material that twinkles and has measurable surface roughness and porosity characteristics that provide a platform for exploring the association between measurable surface roughness and twinkling and for ultimately understanding the physics of twinkling. A “super twinkler” can conceivably be constructed based on macroscopic (tenths of mm) and microscopic (μm) surface roughness features, once the association between surface roughness and twinkling is better understood. Adding an ultrasound twinkling dimension to biopsy marker detection in the care of patients with breast cancer addresses a clinical need and is readily translatable to practice. By building a platform for defining what surface characteristics create and optimize twinkling, commercially available markers could be optimized to twinkle, and 3D printed makers could be made from material readily available and less expensive, such as PMMA.

Current hypotheses on the causes of twinkling have highlighted the presence of air bubbles that vibrate in response to ultrasound insonification and rough surfaces that cause rapid phase changes in the backscattered ultrasound [17–19]. This work does not confirm one hypothesis or the other, and both are supported by the surface roughness observations that we have described.

A twinkling signature associated with a biopsy marker has the potential to improve challenges breast radiologists face during preoperative localization of targets that have responded well to neoadjuvant therapy and are now radiologically normal or occult. While color Doppler ultrasound for detection of twinkling is not a standard part of breast radiology, it is readily available on nearly all cart-based and portable ultrasound vendor platforms. This technological development demonstrates how PMMA with measurable surface roughness features can provide a promising medium to better understand the underlying causes of the twinkling phenomenon on color Doppler ultrasound.

The limitations of using twinkling to detect breast biopsy markers include false-positive entities that twinkle. Sources of false-positive twinkling related to an application in breast radiology include blood flow, microcalcifications [20], calcifications, post-procedural changes with air within soft tissue, and other breast biopsy markers. Careful attention to clinical history and information provided from other imaging modalities can likely distinguish the sources of twinkling. Another limitation of this study is the use of equipment from a single ultrasound vendor. Given the prevalence of the twinkling artifact described on various vendors in the literature, this limitation can likely be readily addressed through vendor-specific equivalents.

Future work will involve creating biopsy markers that are “super twinklers” by refining their surface roughness. In so doing, the underlying causes of ultrasound twinkling may be better understood.

## Data Availability

All data generated or analyzed during this study are included in this published article and its supplementary information files.

## Abbreviations

3D: Three-dimensional
PMMA: Polymethyl methacrylate
*S*_*a*_: Areal surface roughness
SEM: Scanning electron microscopy
